# CICU Care of ACHD Patients: Finally, Some Data to Guide Us

**DOI:** 10.1016/j.jacadv.2024.101076

**Published:** 2024-07-24

**Authors:** William R. Miranda, Nandan S. Anavekar

**Affiliations:** Department of Cardiovascular Diseases, Mayo Clinic, Rochester, Minnesota, USA

**Keywords:** adult congenital heart disease, cardiac intensive care unit, critical care

**Figure:**
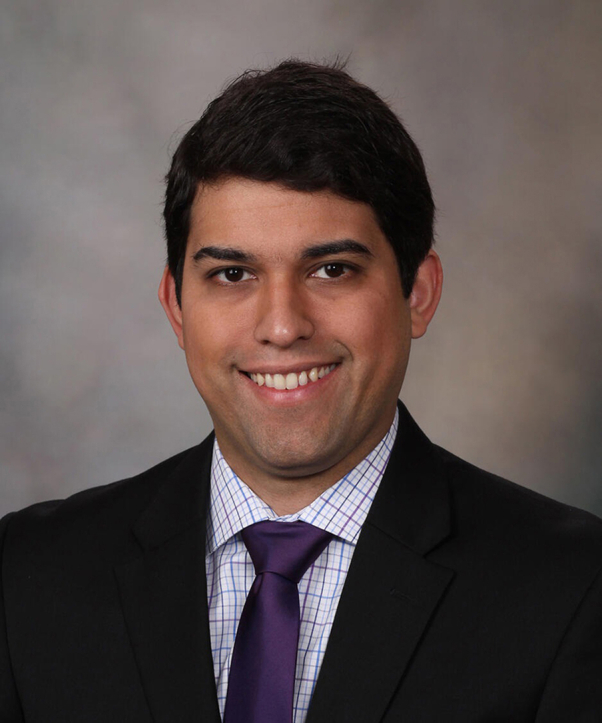

Decades have passed since the inception of cardiac intensive care units (CICUs). In addition to the major impact on the care of ischemic heart disease, CICUs have transformed the management of advanced heart failure, including cardiogenic shock, and electrophysiology emergencies. As our population ages, more than ever, CICU care now goes far beyond cardiovascular disorders, with acute noncardiovascular issues, such as sepsis, prompting, or co-occurring during cardiac emergencies. Lastly, intensive care has never been as tailored, and their outcomes as well-studied. In contrast to this enlightenment, the characteristics and outcomes of individuals with adult congenital heart disease (ACHD) requiring CICU care remain unknown. This knowledge gap is critical since the number of patients with ACHD will inevitably continue to rise.

The contemporary practice of congenital heart disease has a long history of collaboration and innovation, culminating in survivorship in once-considered nonsurvivable lesions. The repair of congenital heart disease is never a correction of the anatomic and physiologic problem and, with survival into adulthood, there is an emergence of presentation of chronic hemodynamic and electrical disturbances that may trigger acute and critical presentations. The challenges in the management of individuals with ACHD are deepened by a lack of evidence-based recommendations for most of the care we provide. This is in stark contradistinction to the current era of randomized controlled clinical trials and meta-analyses that drive the management of more common clinical scenarios, including coronary artery disease and heart failure. To this day, the management of ACHD patients largely remains expert opinion-dependent—perhaps not a big problem at 2 PM but indeed a major issue when an ACHD patient is admitted to CICU at 2 AM. Compound that with the fact that having an ACHD provider at one’s disposal 24/7 is a luxury that only a few institutions can afford, which further emphasizes a potential disparity in health care for this group of patients.

To guide ACHD and non-ACHD providers alike, literature regarding the characteristics and outcomes of ACHD patients requiring CICU care is now overdue. To address this necessity, Keane et al^1^ report data from Critical Care Cardiology Trials Network in ACHD patients admitted to the CICU and compare them to those without an ACHD diagnosis. The authors should be commended for the novelty of their endeavors and findings, as this constitutes a very important step forward for the fields of ACHD and intensive care. As the authors demonstrate—ACHD patients accounted for 2% of CICU admissions—the exposure to the CICU environment if you are an ACHD provider and to ACHD patients if you are an intensivist will happen whether you like it or not.

## Demographics and anatomical diagnoses

As expected, ACHD patients were much younger than those without an ACHD diagnosis, with a median age of 46 years (IQR: 33-60 years). Albeit intuitive, these numbers provide important information regarding the profile of ACHD patients currently admitted to the CICU—they indicated that 25% of individuals were older than 60 years old. This offers insights regarding their potential admitting diagnoses and underlying anatomical defects. It would be unlikely for patients post-Fontan palliation, unrepaired single ventricle, or systemic right ventricles post-atrial switch to fall in this age bracket. Conversely, it would be conceivable that, in a significant proportion of patients having simple congenital cardiac defects repaired in childhood, the current issues were related to superimposed acquired heart disease, for example, coronary artery disease or heart failure with preserved ejection fraction. Collectively, these findings reinforce the importance of ACHD providers being facile with the care of older adults and inherent age-dependent comorbidities.

As commonly seen in clinical practice and in large studies focusing on congenital heart disease, there was marked heterogeneity within the cohort. Shunt lesions constituted the most common anatomical subgroup. However, even in the “simpler group,” phenotypes can markedly vary—ranging from a small, insignificant ventricular septal defect or repaired patent ductus arteriosus to a patient with partial atrioventricular canal with complex surgical and medical history. Similarly, the natural histories and expected comorbidities are significantly discordant among defects, even within the more complex group. These unique aspects of ACHD are critical to the collaboration between intensive care and congenital heart providers so that their joint expertise is converted into individualized and optimal patient care.

## Clinical features

The Sequential Organ Failure Assessment (SOFA) score is a widely used and well-validated tool for the assessment of multiorgan dysfunction in critically ill patients.^2^ This score incorporates ventilatory (PaO_2_, FiO_2_), hemodynamic (mean arterial pressure and need for vasopressor therapy), neurologic (Glasgow Coma Scale), hematologic (platelet count), and hepatorenal (bilirubin and creatinine) function. In addition to providing an assessment of disease severity, it provides prognostic information as increases in score are associated with exponential rises in mortality.

Recently, there has been growth in publications highlighting the prevalence of hepatorenal dysfunction in non-Fontan ACHD patients. This is particularly prevalent in those with right-sided disease, such as those with Ebstein anomaly^3^ and pulmonary atresia with intact ventricular septum.^4^ Moreover, varying degrees of cyanosis are common in this population, being related to residual right-to-left shunt and/or ventilation-perfusion mismatch (restrictive lung disease, diaphragmatic paralysis, etc). Accordingly, these factors might be present even prior to their admission to the CICU. Thus, it is unsurprising that SOFA scores were higher in patients with ACHD. Another cardinal rule of ACHD deserves mentioning here—always get as much historical medical information as you can since things might not have changed as much as you think.

It is also noteworthy that observed mortality rates among ACHD patients were much lower in otherwise predicted by the SOFA scores.^5^ In clinical practice, it is mandatory that we acknowledge the limitations in applying tests and clinical scores derived from non-ACHD patients to those with congenital disorders. These pitfalls are paramount as we make decisions regarding management (ie, how conservative or aggressive to be) and how to counsel families. Therefore, it is mandatory that we remain critical as we interpret these now automatically calculated scores. Lastly, these observations are a perfect set-up for future studies focused on developing ACHD-specific scores of acute cardiovascular illness and prediction of outcomes.

## Reasons for admission and outcomes

The first and third most common reasons for admissions to the CICU were heart failure and atrial arrhythmias. These findings are intuitive. Perhaps counterintuitively, the second most frequent cause for CICU care was general medical problems, accounting for almost one-sixth of patients. This observation can be interpreted in 2 different ways—noncardiac issues are indeed the driver for these admissions. A good example would be a viral respiratory infection disturbing a fine clinical balance. However, in older ACHD patients or in those with complex congenital heart disease, end-organ damage is almost ubiquitous. Therefore, the distinction between cardiac and noncardiac reasons for respiratory, hepatic, and renal insufficiency can be exceedingly difficult, if not impossible. Therefore, from a clinical standpoint, this distinction might be a semantical one.

It is encouraging to see that CICU-related mortality was lower in ACHD patients compared to those without congenital heart defects. However, their CICU stay was much longer and there was no between-group difference in in-hospital mortality. Therefore, providers should not lower their guard after ACHD patients have been transferred out of the unit. In addition, despite their young age, approximately 30% of patients were not dismissed home, underscoring the morbidity associated with a CICU admission in the ACHD population.

## Future steps

Again, the authors should be congratulated for their contribution^1^ and for breaking the inertia into evidence-based CICU care for ACHD patients. Now is the time for the ACHD community to build upon it. Collaboration between ACHD centers has advanced our understanding of other clinically challenging clinical scenarios (transplantation of adults post-Fontan being the most recent one^6^). Such an endeavor would give us a more in-depth analysis of their underlying anatomy, clinical presentation, echo-Doppler data (available in only a minority of patients in the present cohort), and outcomes. Finally, ACHD-specific disease severity and mortality predictors could be devised, thus addressing the need for a clinical score geared to this unique patient population.

## Funding support and author disclosures

The authors have reported that they have no relationships relevant to the contents of this paper to disclose.
